# Evaluation of the Prevalence of Occult Fibrin in Donor Organs, Its Origins, and Consequences: Insights From the COPE Studies

**DOI:** 10.3389/ti.2026.15547

**Published:** 2026-03-25

**Authors:** Christopher J. E. Watson, Ina Jochmans, Stephen Macdonald, Danielle White, Christopher Bridgeman, Andrew J. Butler, Rohit Gaurav, Lisa Swift, Anna L. Paterson, Letizia Lo Faro, Maria E. Kaisar, David Nasralla, Peri Husen, Sarah Cross, Peter J. Friend, Rutger J. Ploeg, Vasilis Kosmoliaptsis

**Affiliations:** 1 Department of Surgery, Addenbrookes Hospital, University of Cambridge, Cambridge, United Kingdom; 2 The Roy Calne Transplant Unit, Cambridge University Hospitals NHS Foundation Trust, Cambridge, United Kingdom; 3 National Institute for Health and Care Research, Cambridge Biomedical Research Centre, Cambridge, United Kingdom; 4 National Institute for Health and Care Research Blood and Transplant Research Unit in Organ Donation and Transplantation, at The University of Cambridge, in Collaboration With Newcastle University, in Partnership With National Health Service Blood and Transplant (NHSBT), Cambridge, United Kingdom; 5 Laboratory of Abdominal Transplantation, Transplantation Research Group, Department of Microbiology, Immunology and Transplantation, KU Leuven, and Abdominal Transplant Surgery, University Hospitals Leuven, Leuven, Belgium; 6 Specialist Haemostasis Laboratory, Cambridge University Hospitals NHS Foundation Trust, Cambridge, United Kingdom; 7 Department of Histopathology, Cambridge University Hospitals NHS Foundation Trust, Cambridge, United Kingdom; 8 Oxford Transplant Research Group, Nuffield Department of Surgical Sciences, John Radcliffe Hospital, University of Oxford, Oxford, United Kingdom; 9 Liver Transplant Unit, The Royal Free London NHS Foundation Trust, London, United Kingdom; 10 Department of General, Visceral and Transplant Surgery, University Hospital Heidelberg, Heidelberg, Germany; 11 Nuffield Department of Surgical Sciences, University of Oxford, and The Oxford Biomedical Research Centre, Oxford, United Kingdom

**Keywords:** D-dimers, machine perfusion, microthrombi, organ donation, transplantation

## Abstract

Microthrombi are often observed in the glomerular tufts of peri-transplant renal biopsies, and occult fibrin has been described in livers undergoing normothermic perfusion, with its presence associated with cholangiopathy and poorer transplant survival. To further examine the phenomenon, we measured D-dimers in the perfusates of kidneys and livers that were part of organ perfusion studies conducted by the Consortium for Organ Preservation in Europe. Both kidneys and livers were found to contain variable amounts of D-dimers. The need for dialysis in kidneys donated after circulatory death (DCD) was associated with higher levels of D-dimers in the hypothermic kidney perfusate. Higher amounts of D-dimers in the liver perfusate were associated with poorer liver transplant survival. There was no significant difference in D-dimer release from livers and kidneys between donors who died from head trauma, stroke, or hypoxia. Organs from donors who died by euthanasia had significantly fewer D-dimers. This study shows that occult fibrin is common in both livers and kidneys from deceased donors and has adverse consequences. The different D-dimer loads by donor cause of death suggest a donor origin for at least some of the occult fibrin.

## Graphical Abstract

**Figure:**
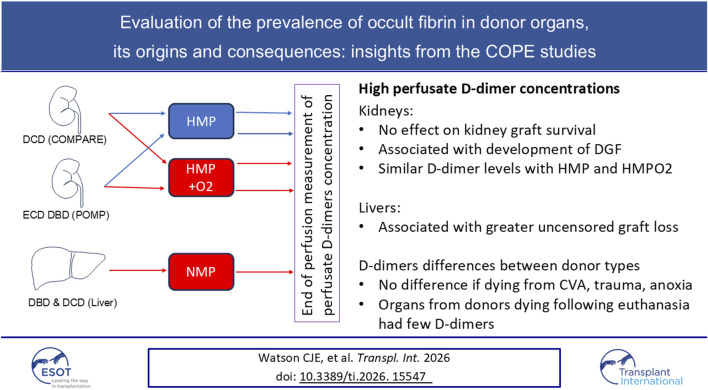

## Introduction

It has long been recognized that donor kidneys may harbor fibrin microthrombi, most commonly observed within the glomerular tufts of early renal biopsies. However, their origin, significance, and prevalence are not clear. Early reports have suggested a recipient origin related to ciclosporin [1], while more recently they have been suggested to be of donor origin or to develop during cold storage; their significance has also been questioned [2–6]. Studies of livers undergoing end-ischemic *ex situ* normothermic machine perfusion (NMP) have also revealed the presence of microthrombi, which manifest as D-dimers in the perfusate [7].

D-dimers are breakdown products of polymerized fibrin. The D-dimer assay has a high sensitivity but low specificity for thromboembolic disease, as it is elevated in other conditions, such as cancer and infection. It is likely that, even in these conditions, an elevated D-dimer level signals the presence of fibrin polymer breakdown products. The test is simple to perform and is in routine use for the diagnosis of thromboembolic disease, where a negative test result excludes venous thrombosis or embolism. In the context of *ex situ* organ perfusion, the presence of D-dimers is highly likely to represent occult fibrin being flushed out of the organ.

The release of large amounts of D-dimers into the liver perfusate has been associated with a higher incidence of post-transplant cholangiopathy and poorer transplant survival compared to livers with less D-dimer release, and by extrapolation, less occult fibrin [7]. Fibrin plugs in the peribiliary vascular plexus, coinciding with areas of stromal necrosis, have been noted in livers donated after circulatory death (DCD) that underwent brief periods of NMP [8], supporting the causative role of fibrin in transplant cholangiopathy. D-dimers have also been observed to be released during hypothermic liver perfusion [7, 9]. These observations raise several questions:

1. Are D-dimers in the NMP perfusate associated with adverse outcomes in other series of perfused livers? If so, are they present in livers undergoing NMP from the point of recovery at the donor hospital, even with minimal exposure to cold ischemia?
2. What is the prevalence of D-dimers/fibrin in organs other than the liver, and if so, are they a marker of poor outcomes?
3. If fibrin originates in the donor, is its prevalence affected by donor cause of death?

In an attempt to address these questions, we sought samples from the biorepository created as part of the Consortium for Organ Preservation in Europe (COPE), a European Union Seventh Framework Programme (FP7)-funded research program that includes three preservation studies where perfusate samples of both livers and kidneys were taken during machine perfusion and stored for future analysis [10–12]. In addition, we looked at our own data on D-dimers released from livers undergoing NMP, particularly referencing donor cause of death to look for possible associations. Finally, D-dimers were also measured in pre-retrieval blood samples from donors after brain death (DBD) and circulatory death obtained from the Quality in Organ Donation (QUOD) biorepository, whose livers underwent NMP at our institution, to see whether they were present in the donors and, if so, whether their prevalence corresponded to D-dimer load in the liver.

## Materials and Methods

The Consortium for Organ Preservation in Europe (COPE) was an FP7-funded program of research that included two randomized multicenter trials of hypothermic kidney perfusion and one randomized multicenter study of normothermic liver perfusion. The details of these studies have been published elsewhere [10–12] but are summarized below. All three trials had received the appropriate ethics approvals (POMP: 14/SC/1072; COMPARE: 14/SC/1056; and Liver: 14/LO/0182). In the COPE studies, perfusate samples were taken at pre-defined time points, centrifuged, and the supernatant was frozen and transferred to the biorepository at Oxford, where they were thawed once for aliquoting and stored at −80 °C.

### The COPE Cold Oxygenated Machine Perfusion of Aged REnal Transplants (COMPARE) Study

The COMPARE study was a randomized controlled trial in which pairs of kidneys from the same DCD donor were randomized such that one underwent oxygenated hypothermic machine perfusion (HMPO2) and the other underwent non-oxygenated hypothermic machine perfusion (HMP) from the donor hospital until removed for transplantation at the recipient centre using Machine Perfusion University of Wisconsin solution and the XVIVO Kidney Assist Transport [11]. Perfusate samples were taken 15 min after the start of perfusion and at the end of perfusion.

### The COPE Pre-Implantation Oxygenated Hypothermic Machine Perfusion (POMP) Study

The POMP study involved expanded criteria DBD donor kidneys, which were randomized on arrival at the recipient center to either remain stored on ice in their original cold storage solution, or to undergo end-ischemic oxygenated cold perfusion (HMPO2) until removed for implantation [12]. The XVIVO Kidney Assist Transport was also used for this study. Perfusate was collected after 15 min of perfusion and at the end of perfusion and processed as in the COMPARE study.

### The COPE Liver Study

The COPE liver study was a device-to-donor study of the *metra®* (OrganOx, Oxford, UK) that randomized livers from both DBD and DCD donors to either standard cold storage in a University of Wisconsin solution or normothermic machine perfusion on the *metra* from the point of retrieval at the donor hospital until implantation at the recipient center [10]. Samples of perfusate were collected in ethylene diamine tetra-acetic acid (EDTA) after 1 h of perfusion.

### Liver NMP Cases

For additional comparison of D-dimer concentrations released from livers of different deceased donors, we also compared a cohort of NMP liver perfusions undertaken in a back-to-base setting at our own institution. Perfusate samples had been taken after 2 h of NMP, centrifuged, and the supernatant stored at −80 °C. The 2-h time point was chosen for sampling because previous work had suggested it to be a time point after which D-dimer concentrations tended to plateau in some livers; it also corresponded to the previously used time point [7]. Cases receiving tissue plasminogen activator and those that had previously undergone normothermic regional perfusion were excluded.

### QUOD Samples

The Quality in Organ Donation (QUOD) initiative is a biorepository in the United Kingdom that collects blood, urine, and tissue samples from consenting deceased organ donors to facilitate research. Samples include blood taken prior to organ recovery [13]. The pre-retrieval donor D-dimer content was assayed in the last EDTA sample from 78 donors who were selected based on high or low D-dimer levels detected after 2 h of normothermic liver perfusion. These livers included those that had and had not been exposed to alteplase plus fresh frozen plasma thrombolysis. This allowed us to assess the prevalence of fibrin degradation products in the donors and their relation to those in the liver perfusate.

### D-dimer measurement

D-dimers were measured using latex immunoturbidimetry with the D-dimer HS assay on the ACLTOP 750CTS analyzer (Werfen, Barcelona, Spain) according to the manufacturer’s instructions.

### Statistical Analysis

Statistical analysis and graphing were performed using GraphPad Prism version 10.5 for macOS (GraphPad software, Boston, USA). Comparisons between multiple groups were undertaken with the Kruskal-Wallis test, while the Kolmogorov-Smirnov test was used for comparisons between two groups, since not all groups were normally distributed. Survival was compared with the Log-rank test. The Wilcoxon matched pairs test was used to compare pairs of kidneys from the same donor with different treatments.

## Results

Not all organs in each of the three COPE trials had perfusate samples taken, nor were all perfused organs transplanted.

### D-dimers in COMPARE Study Kidneys

The COMPARE study included 112 samples taken 15 min after perfusion and 157 samples taken at the end of perfusion (median duration of perfusion: 547 min; IQR: 392–691), including 68 kidneys with samples taken at both time points. Of those 68 kidneys, there were more D-dimers present in the perfusate at the end of perfusion (median: 878 ng/mL; IQR: 335–2069) than at 15 min (median: 91 ng/mL; IQR: 52–217), with a close correlation between the samples taken at 15 min and at the end of perfusion (Spearman r = 0.7826; p < 0.0001; Supplementary Figure S1).

**FIGURE 1:**
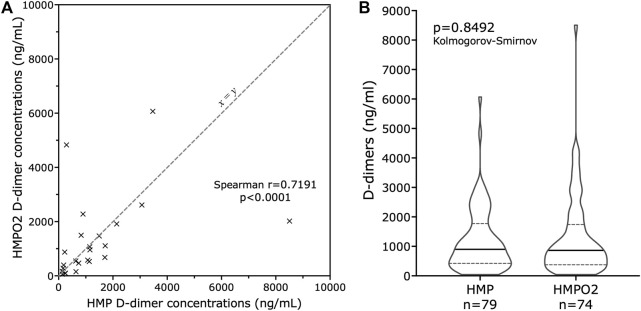
D-dimer concentrations in kidneys undergoing HMP and HMPO2 in the COMPARE study. Panel **(A)** shows the D-dimer concentrations in the perfusate at the end of perfusion in 27 pairs of DCD donor kidneys from the same donor, where one kidney underwent HMP with oxygen (HMPO2) and one kidney underwent HMP without. There is a strong correlation (r = 0.731) between the two D-dimer concentrations. The dashed line is the line of equivalence (x = y). Panel **(B)** is a violin plot showing the D-dimer concentrations at the end of perfusion for all kidneys undergoing HMPO2 and HMP, with no significant difference between the groups (p = 0.885). The horizontal dashed lines (- -) represent the interquartile range, and the solid line (—) represents the median values.

There were also samples from 24 pairs of kidneys from the same donor, one of which had undergone HMPO2 and the other HMP alone from the time of retrieval. End-of-perfusion D-dimer concentrations were closely correlated (Spearman r = 0.7191, p < 0.0001; Figure 1A), with no significant difference between groups (p = 0.4223, Wilcoxon matched pairs). Overall, of the 157 end-of-perfusion samples, 74 were from kidneys that underwent HMPO2, and 79 were from kidneys that underwent HMP alone. Two other kidneys received unspecified interventions, and two were described as having received cold storage alone but with perfusate samples; the true nature of these last two is unclear, and they were excluded from our analysis. There was no difference in D-dimer concentrations between the end of perfusion samples for HMPO2 and HMP kidneys (p = 0.8847, Figure 1B), which also suggests that oxygenating the perfusate did not affect D-dimer concentrations.

**FIGURE 2:**
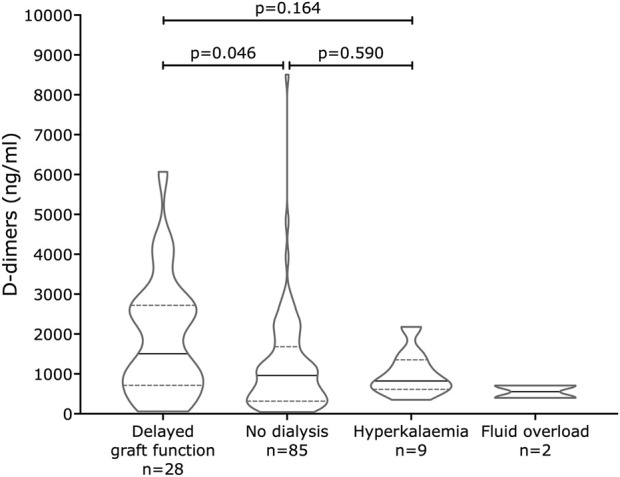
Violin plots of end-of-perfusion D-dimer levels in COMPARE kidneys by early graft function classification. There were significantly greater D-dimer concentrations at the end of perfusion in kidneys that were defined as having delayed graft function due to the need for multiple dialysis episodes compared to those not requiring dialysis. In this truncated violin plot, the horizontal dashed lines (- -) represent the interquartile range, and the solid line (—) represents the median values.

Investigators had classified the need for dialysis in the first week post-transplant into four groups: no dialysis (n = 85); delayed graft function (DGF, n = 28); hyperkalemia (a single post-transplant dialysis due to high serum potassium, n = 9); and fluid overload (the need for a single dialysis episode to remove excess fluid, n = 2). Eleven kidneys that failed within the first seven days were excluded, as were 21 kidneys for which follow-up consent was not given. Of the 11 kidneys lost in the first week, 4 were due to technical reasons related to vascular anastomosis, 4 were described as having an immunological cause, 2 had primary nonfunction, and one had recurrent focal segmental glomerulosclerosis. There was a significant difference in D-dimer concentrations between kidneys with and without delayed graft function (p = 0.0461, Kolmogorov-Smirnov, Figure 2).

**FIGURE 3:**
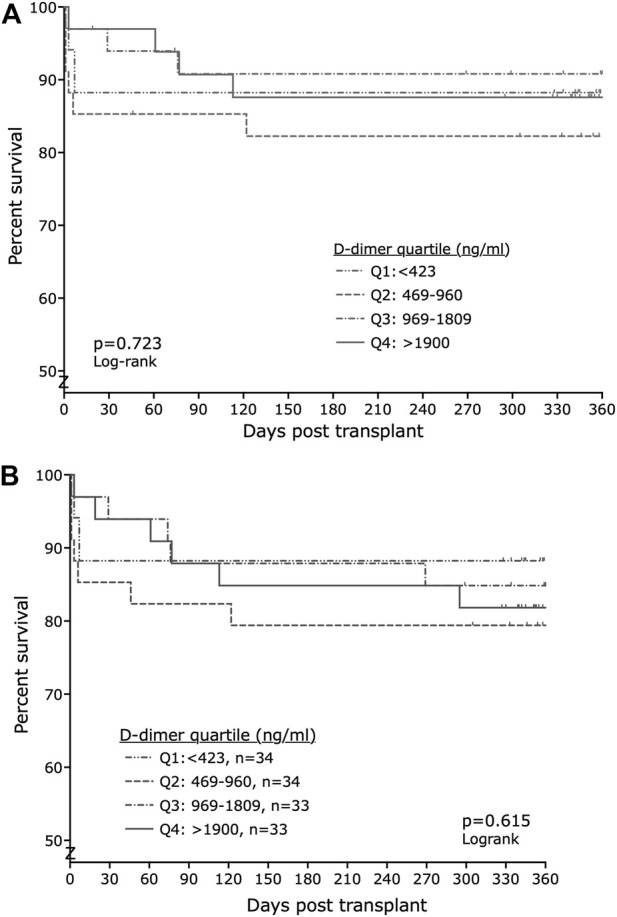
There was no significant difference between groups in either **(A)** graft survival (censored for death) or **(B)** transplant survival (graft survival not censored for death).

There was no significant difference by D-dimer quartile in glomerular filtration rate at 12 months, as calculated from the Chronic Kidney Disease Epidemiology Collaboration (CKD-Epi) formula (data not shown). Graft survival was characterized by more early graft loss within 30 days in the two quartiles with the lowest D-dimer concentrations and more late graft loss in the quartiles with the higher D-dimers, but overall there was no significant difference in graft or transplant survival (for graft survival p = 0.7234, for transplant survival p = 0.6147, logrank test; Figures 3A,B).

### D-dimers in POMP Study Kidneys

The POMP study included 74 perfusate samples taken 15 min after the start of perfusion and 74 samples taken at the end of perfusion (median: 289 min; IQR: 188–439), including 71 kidneys for which both beginning and end of perfusion samples were available. There was a statistically significant, moderate correlation between the 15-minute and end-of-perfusion samples (Spearman r = 0.5857, p < 0.0001, Supplementary Figure S2), with the end-of-perfusion samples containing more D-dimers. There was also a significant, moderate correlation between the duration of end-ischemic HMPO2 and D-dimer concentrations (Spearman r = 0.4443, p = 0.0001, Supplementary Figure S3). In the POMP study, the need for dialysis was classified as due to delayed graft function (n = 16), fluid overload (n = 1), other (n = 1), or no dialysis required (n = 56) (Supplementary Figure S4). There was no correlation between delayed graft function and end-of-perfusion D-dimer concentrations.

Only three of the kidneys for which perfusate was available were lost during the study period. These included the kidney with the highest recorded D-dimer concentration (3,875 ng/mL), which was lost to venous thrombosis on day 8.

The median 15-minute perfusate D-dimer concentration in the COMPARE study was slightly higher than that in the POMP study (COMPARE: 90 ng/mL, IQR 52 to 217; POMP: 65 ng/mL, IQR 50 to 109, p = 0.0168). The end-of-perfusion perfusate D-dimer concentrations were much higher in the COMPARE study than in the POMP study (COMPARE median 878 ng/mL, IQR 334–2069; POMP 300 ng/mL, IQR 174–556, p < 0.0001). When interpreting these data, it is important to remember that the COMPARE kidneys were from DCD donors, while the POMP kidneys were from extended criteria DBD donors, and that the duration of perfusion was longer in the COMPARE study than in the POMP study (median duration in the COMPARE study was 547 min vs. 289 min in the POMP study).

### D-dimers in the COPE Liver Study

**FIGURE 4:**
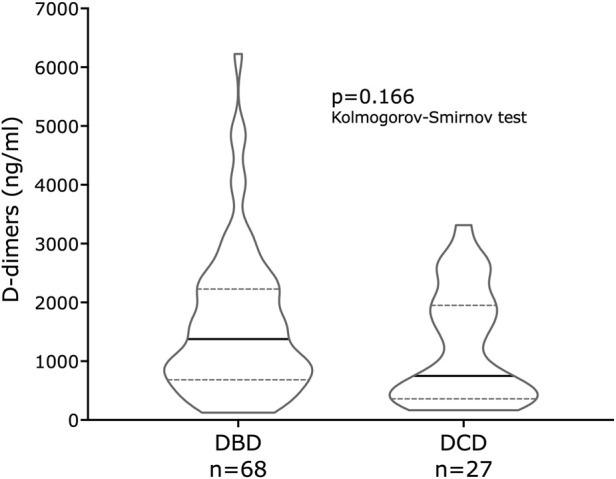
D-dimer concentrations in the COPE liver machine perfusion study. Panel 4 is a violin plot of D-dimer concentrations after 1 h of perfusion in DBD and DCD donor livers, where the horizontal line represents the median value and the dotted lines represent the interquartile ranges. There is no significant difference in D-dimer concentrations between DBD and DCD donor livers.

Perfusion samples were collected 1 h after the start of perfusion from 95 livers (68 from DBD donors and 27 from DCD donors). The median D-dimer concentration was 1,376 ng/mL (IQR: 684–2228) for DBD livers and 750 ng/mL (IQR: 360–1950) for DCD livers, a difference that was not significant (Figure 4; p = 0.1657, Kolmogorov-Smirnov).

**FIGURE 5:**
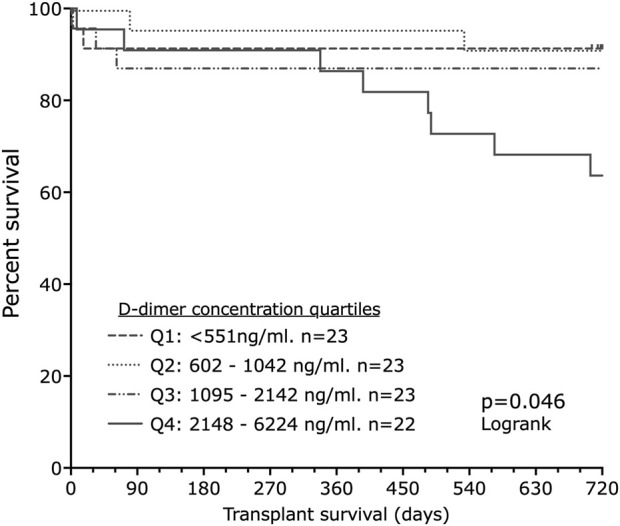
Liver transplant survival by D-dimer quartile. There is a significant increase in transplant loss (death or graft failure) at 2 years with increasing D-dimer quartiles (Log-rank p = 0.046).

Of the 95 livers for which samples were received, 91 were transplanted. Two-year follow-up data were available for all transplanted livers. The transplanted livers were divided into quartiles based on D-dimer concentrations. Transplant failure (graft loss (n = 2) or death (n = 13)) was more likely in the quartile with the highest D-dimer concentration (p = 0.0458, Logrank, Figure 5).

Of the livers for which a perfusate sample was obtained, 59 had undergone magnetic resonance cholangiopancreatography (MRCP) at 6 months. None of these livers had definite cholangiopathy affecting the peripheral ducts; however, three had hilar strictures, which may have been secondary to cholangiopathy or related to the placement of the bile cannula before perfusion; the D-dimer concentrations of these three livers were 246, 669, and 1,950 ng/mL, respectively.

### D-dimers by Cause of Death

**FIGURE 6:**
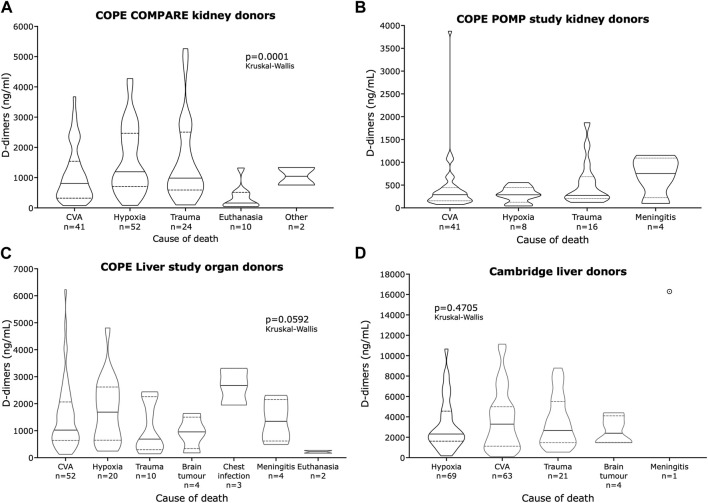
Violin plots of four groups of organs and their D-dimer concentrations by cause of donor death. **(A)** End of perfusion samples in the COMPARE study; **(B)** End of perfusion samples in the POMP study; **(C)** One-hour perfusate samples in the COPE liver study, and **(D)** 2-h perfusate samples in the investigators’ liver NMP cohort.

D-dimer concentrations were examined by donor cause of death in organs from the COPE liver study, the COMPARE and POMP kidney studies, and a cohort of perfused livers from our own center (Figure 6). There was no difference observed in D-dimer concentrations between donors who died from cerebrovascular accidents (CVAs), hypoxia, or trauma in any of the study populations. Both the COMPARE kidney study and the COPE liver study had separate donor cohorts who died from euthanasia, and organs from those donors were associated with significantly lower D-dimer concentrations than organs from donors who died from other causes. For example, in the COMPARE study, donors who died following euthanasia had significantly lower D-dimers than donors who died from hypoxia (p < 0.0001), trauma (p < 0.0003), or cerebrovascular accidents (p = 0.002, all tested using the Kolmogorov-Smirnov test). In contrast, donors who died of meningitis tended to have higher D-dimer concentrations. The COPE liver study also included three donors described as having died of chest infections who also had high D-dimer concentrations.

### D-dimers in QUOD Donors

Pre-donation samples were obtained from 77 liver donors whose livers subsequently underwent NMP at our institution; all had pre-donation D-dimer levels above the normal range (<250 ng/mL), suggesting the presence of intravascular thrombus breakdown products in their circulation, with donors who died from trauma having the highest D-dimer concentrations (Supplementary Figure S5). DBD donors had higher D-dimers than DCD donors (Supplementary Figures S5, S6, p = 0.046, Kruskal-Wallis), but this was a selected group of donors based on liver D-dimer release; therefore, no inference about differences between DBD and DCD donors should be made. There was no significant correlation between D-dimer concentrations in donors and D-dimer concentrations in the liver perfusate of TPA-treated (n = 37) or untreated (n = 40) livers.

## Discussion

In this study, we demonstrated that the release of D-dimers from organs of deceased donors during *ex situ* perfusion is common and occurs in both kidneys and livers. In addition, we confirmed the previous observation from the Cambridge study that the presence of high concentrations of D-dimers in livers is associated with increased transplant failure (graft loss not censored for death). Unlike the Cambridge study, the COPE study livers were placed on NMP at the donor hospital, with very short periods of cold storage, suggesting that the fibrin formation is probably not secondary to cold storage alone, although a contribution from cold storage cannot be ruled out. The D-dimer concentrations were also much lower in the COPE livers than in Cambridge livers. While this may reflect a contribution of cold storage-induced fibrin in the Cambridge livers, it is also possible that the shorter period of NMP before samples were taken (1 h vs. 2 h) may have meant there was less time for flushing out the vasculature.

We also demonstrated that D-dimer concentrations increased with time in both kidney cohorts, being lower after 15 min of perfusion and higher at the end of perfusion, and that there was a correlation between the early and late perfusion D-dimers, supporting the notion that long hypothermic perfusions are associated with greater washout of pre-existing intravascular fibrin. This is further supported by the observation that the extended criteria DBD kidneys undergoing oxygenated perfusion at the recipient center in the POMP study tended to release fewer D-dimers than DCD kidneys undergoing HMP or HMPO2 from the donor hospital in the COMPARE study, during which the duration of perfusion was much longer (the median duration in the COMPARE study was 547 min vs. 289 min in the POMP study). It is also possible that this difference relates to a greater burden of fibrin in the DCD donor kidneys in the COMPARE study, or both factors may be at play.

There was no difference in D-dimer concentrations in the COMPARE study between kidneys undergoing HMP and those undergoing HMPO2, suggesting that oxygenation during hypothermic perfusion does not affect the prevalence of fibrin. Kidneys defined as having delayed graft function appeared to have higher D-dimer loads than kidneys categorized as needing dialysis for hyperkalemia or fluid overload, supporting the researchers’ differing indications for dialysis. The D-dimer load did not translate into poorer graft survival.

The difference in D-dimer amounts in organs according to the cause of death of the organ donor was also notable. There was no significant difference in D-dimer release in all the studies between donors who died from stroke (cerebrovascular accident), trauma, or hypoxia; however, there was a significant lack of D-dimers in donors who died by euthanasia. It is unclear whether this relates to treatment at the time of donation [14–16] or to the absence of a circulatory insult in the time leading up to death, following which a clot forms while hospitalized or as a result of stress. Further investigation is required. Donors undergoing normothermic regional perfusion were excluded from the study due to prior evidence showing an association between this procedure and low levels of D-dimer release [7]. In the absence of NRP, we demonstrated here that DCD donor organs shed equivalent amounts of D-dimers as DBD livers.

The subgroup of kidneys in the COMPARE study from euthanized donors was reported to have lower vascular resistance during perfusion compared to other kidneys in that same study [17] and it is possible that the absence of microthrombi may contribute to this observation and explain the variable resistance seen during HMP of donor kidneys in general. This is the subject of investigation by other researchers.

Another observation, based on a small number of cases, is that organs from five donors who died from meningitis and three donors with active chest infections due to chronic pulmonary disease appeared to release higher concentrations of D-dimers, suggesting a contribution of donor sepsis to their etiology. This observation requires substantiation in a larger cohort.

All organ donors in the QUOD study had abnormally high concentrations of D-dimers, possibly reflecting the cause of death, such as trauma; it may also reflect thromboembolic complications in the intensive care unit. The lack of correlation between D-dimers released from the liver during NMP and those in the donor’s blood suggests that screening donors to identify at-risk organs would not be worthwhile. This and other observations pose a challenge for neuro-intensivists, since disseminated thrombosis appears to be common, and its contribution to neurological outcomes is uncertain.

Thrombi are often observed in the glomeruli of deceased donor kidneys [4, 5, 18], but their prevalence has hitherto been unknown. A recent study of over a thousand pre-implantation and implantation punch biopsies revealed a <2% incidence of microvascular thrombi, the majority of which had no thrombotic clinical sequelae [19]; this is consistent with other studies. Nevertheless, sampling glomeruli may miss thrombi in peritubular capillaries. Occlusion of these capillaries and of the arteries within the glomeruli may contribute to acute tubular necrosis. Our data suggest that fibrin microthrombi are common and that they may have an etiological role in DGF. They may contribute to the different degrees of resistance during hypothermic perfusion. These microthrombi, together with vascular spasm, probably contribute to the blotchy appearance of the kidneys during reperfusion, during which the slow flow of arterial blood results in desaturation, with a resultant blue hue to the kidney until the prevailing arterial pressure manages to clear some of the occluding microthrombi.

Our data, particularly the observations relating to donors who died as a consequence of euthanasia, provide strong evidence to support the fact that at least some of the fibrin originates in the donors before donation. These data align with observations that thromboelastography of both DCD and DBD donor blood before retrieval is hypercoagulable [20]. We have not been able to determine whether organs also acquire fibrin microthrombi during cold storage, something that would contribute to the adverse effects of extended periods of cold ischemia. Nevertheless, the prevalence of microthrombi in donor organs suggests attention should be paid to the management of patients with severe neurological injury to minimise the risk of thrombi formation in all organs.

In this article, we have shown that hypothermic machine perfusion does flush out fibrin fragments, and previous reports have confirmed the same observations with hypothermic oxygenated liver perfusion (HOPE) [7, 9]. The relatively high incidence of cholangiopathy following HOPE in the European randomized trial, 14% at 5 years, may reflect the relatively short perfusion period [21]. Flushing out fibrin fragments probably does contribute to superior kidney outcomes with prolonged preservation using HMP compared to static cold storage alone [22].

An alternative treatment is *ex situ* normothermic perfusion with recombinant tissue plasminogen activator (TPA) and a plasminogen source. This has been shown to release more D-dimers from livers than normothermic perfusion without thrombolytics [7, 23], but further work is required to define optimal protocols for clinical use, with their efficacy proven in a randomized setting. TPA treatment may also play a role in kidney transplantation, given the association of D-dimers with delayed graft function, and warrants further investigation. When thrombolysis is not undertaken, measuring D-dimers during perfusion may prove a valuable diagnostic tool for the assessment of the viability of deceased donor organs.

A retrospective analysis of prospectively collected samples such as this has limitations. One major limitation is the lack of standardization in the timing of perfusate sampling. Future studies should include serial sampling at standard time points.

In summary, this study confirms the previous observation that occult fibrin in donor livers is associated with adverse graft outcomes and also demonstrates that it is associated with delayed graft function in kidneys. We demonstrated that all of the organ donors studied had high levels of D-dimers, suggesting intravascular fibrin. These levels were not correlated with the levels found in the livers of the same donors. Perfusion, whether hypothermic or normothermic, appears to reduce the fibrin burden and may thus contribute to improved outcomes. Finally, we showed that donors who died by euthanasia have no fibrin burden, in contrast to those donors who died from other causes. The next challenge is to rid the organs of their occult fibrin before it can affect post-transplant outcomes.

## Data Availability

The raw data supporting the conclusions of this article will be made available by the authors, subject to reasonable request.
