# Supplementary material for: Evaluation of the Prevalence of Occult Fibrin in Donor Organs, Its Origins, and Consequences: Insights From the COPE Studies
Source: Transpl Int. 2026 Mar 25;39:15547. doi: 10.3389/ti.2026.15547 (PMC13056880; doi:10.3389/ti.2026.15547)

Supplemental Material

Evaluation of the prevalence of occult fibrin in donor organs, its origins and consequences: insights from the COPE studies.

D-dimers in the Compare study

There is a close correlation between D-dimer concentrations after 15 minutes and at the end of perfusion, with the concentrations at the end of perfusion being much greater (Fig S1).

Figure S1. Graph showing correlation between D-dimers at the start and end of perfusion for kidneys in the COMPARE study

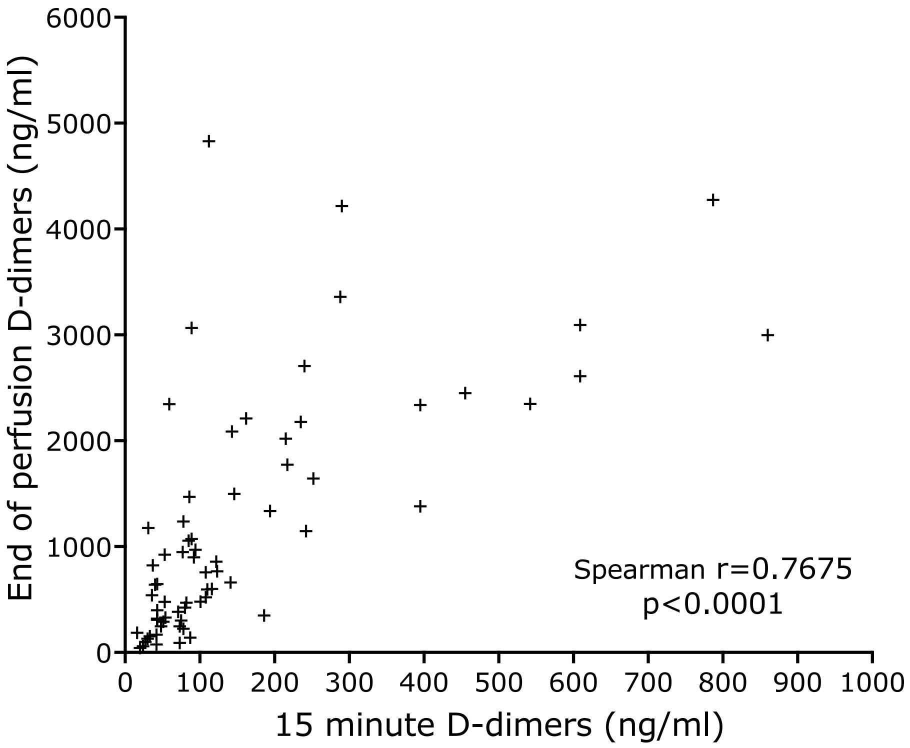

The POMP study

There was a statistically significant moderate correlation between the 15 minute and end of perfusion samples (fig S2). The Spearman correlation index r = 0.5857, p<0.0001.

Figure S2. Graph showing correlation between D-dimers at the start and end of perfusion for kidneys in the POMP study

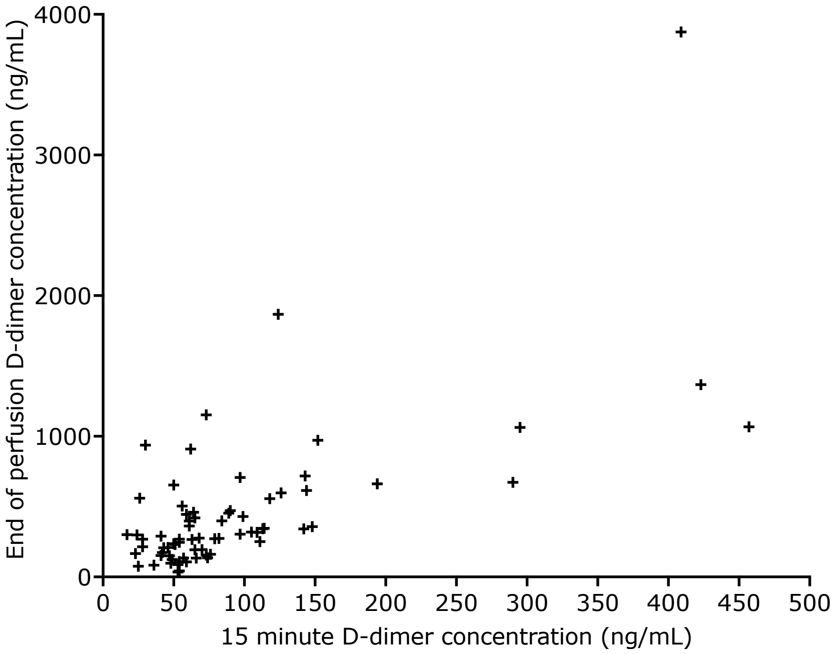

There was also a moderate but significant correlation between duration of the end-ischaemic HMPO2 and D-dimer concentration (fig S3), Spearman correlation coefficient r = 0.4342, p=0.0001

Figure S3. Graph showing D-dimer concentration with duration of perfusion.

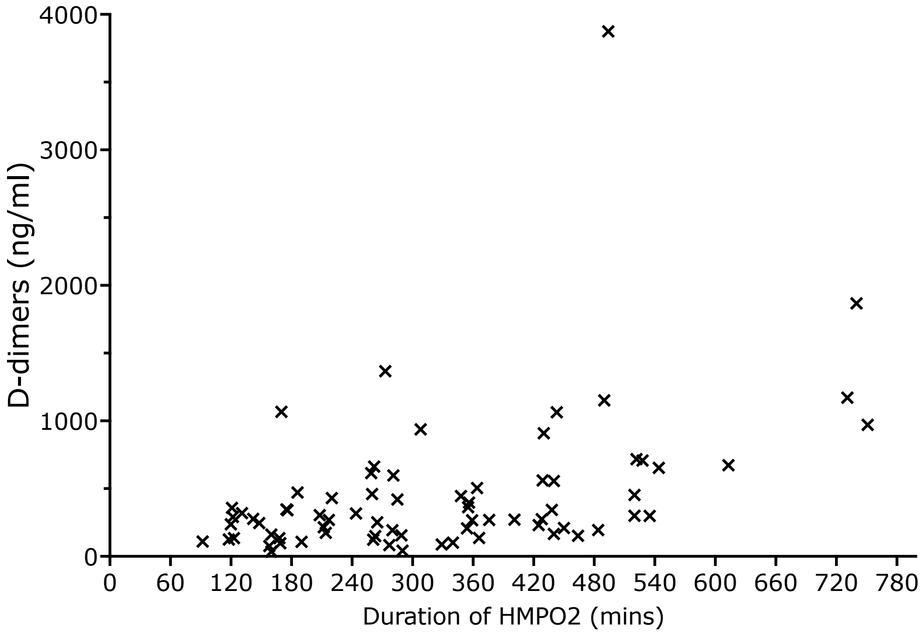

In the POMP study the need for dialysis was classed either as being due to delayed graft function (n=16), fluid overload (n=1), other (n=1) or no dialysis required (n=56). There was no relation between delayed graft function and end of perfusion D-dimer concentration (fig S4).

Figure S4. End of perfusion D-dimer concentration by indication for dialysis in the POMP study

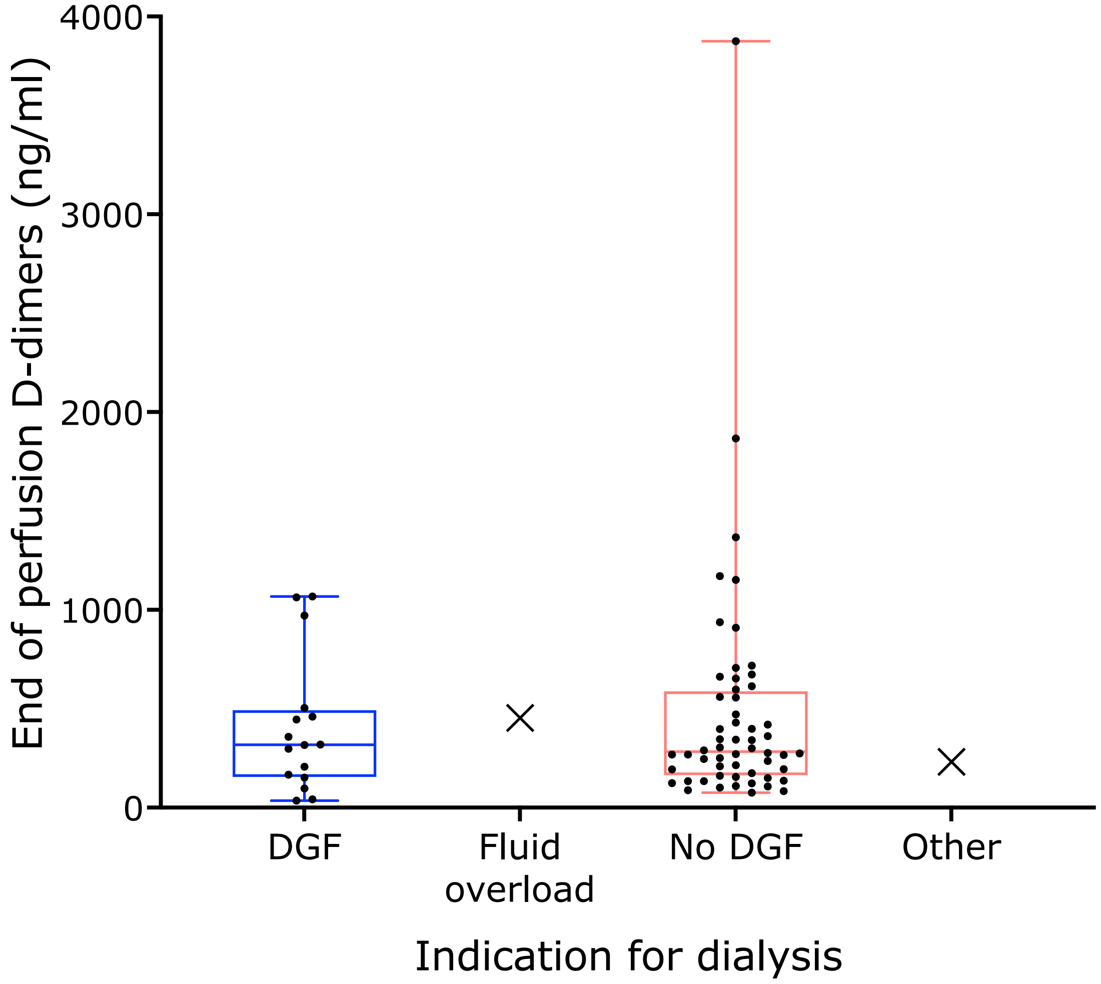

D-dimers in QUOD organ donors

Samples from 77 liver donors were obtained; all had D-dimer levels above the normal range (<250ng/mL) suggesting the presence of breakdown products of intravascular thrombus within their circulation, with donors dying from trauma having the highest D-dimer concentrations.

Supplementary figure S5: D-dimer levels in organ donors by cause of donor death

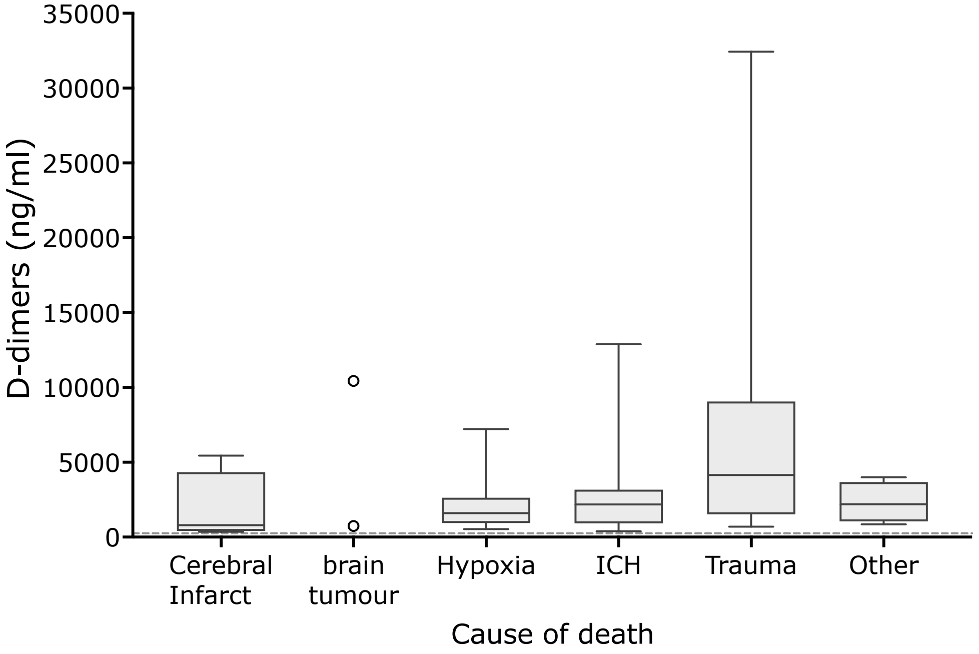

DBD donors had more D-dimers than DCD donors, (fig S6, p=0.046, Kruskal Wallis).

Figure S6: DBD donors had higher levels of D-dimers than DCD levels (p=0.046)

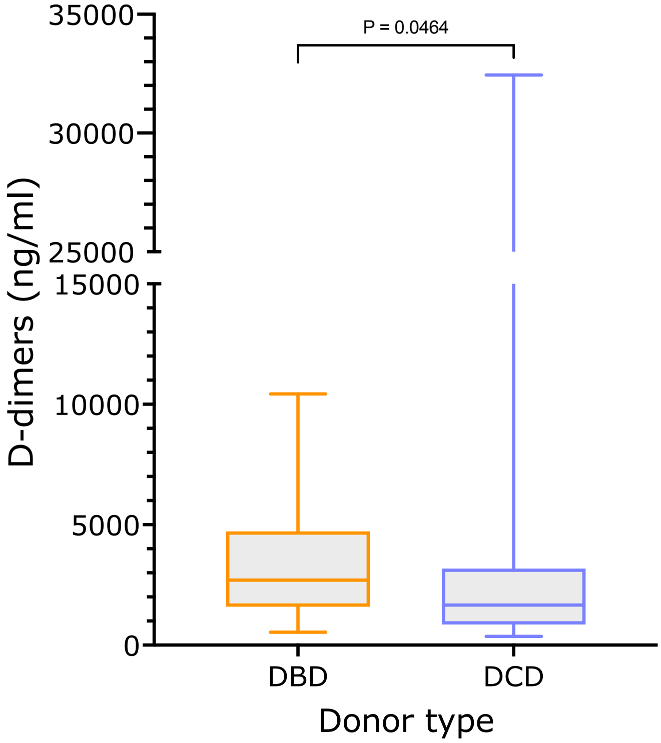

Supplement: Supplementary file 1 [file DataSheet1.pdf]
